# Fracture of Astragalus

**Published:** 1867

**Authors:** Henry T. Godfrey

**Affiliations:** Benton, Wis.


*Fracture of Astragalus.—Dislocation of Foot Inwards.* By HENRY T. GODFREY, M.D., Benton, Wis.

*Aug. 17, 1866.* Was called to visit Mrs. Rob't. R., *æt.* 37, mother of six children, three months pregnant. She had been run away with by a pair of young horses, and had jumped from the wagon, injuring her right foot.

Upon examination, I found a dislocation of the right foot inwards; the superior surface resting against the internal edge of the tibia, and the sole of the foot looking towards the opposite limb. The fibula was fractured, about three inches above the ankle. The upper articular surface of the astragalus formed a tumor below and in front of the external maleolus. Attempts at reduction had been made by a "bone-setter," and had not at all tended to the patient's comfort.

Having placed the patient in bed, administered chloroform to complete anæsthesia, and instructed an assistant to hold the knee firmly in a semi-flexed position, made extension with the hand, at the same time pressing upon the displaced portion of astragalus to effect its reduction. The foot was easily restored to its position by these means, but, after repeated efforts, I abandoned the attempt at reduction of the fractured astragalus as impossible. According to the opinion of Mr. Hancock, expressed in his valuable lectures on the anatomy and surgery of the foot, section of the tendo-Achillis and, perhaps, also of the tendons of the tibiali muscles, if they seemed to obstruct reduction, would have been the most appropriate treatment. I did not adopt this plan, because there is some risk of the retraction of the tendons and the loss of power of their muscles; because in dislocation of the head of a bone from its place, the ligaments offer the greatest amount of resistance to reduction, and the resistance can be sufficiently overcome by chloroform; and, lastly, because the amount of violence necessary to cause a fracture of this bone is very great, and necessarily followed by severe inflammation, which would be much heightened by such operations. I decided to allow the bone to remain where I found it, and if it produced sufficient irritation to demand it to practice excision. Placed the leg in a fracture box, well padded, and directed cold water to be applied. Gave ½ grain morphine every four hours.

*Aug. 26.* Patient has progressed quite favorably till this time. Found her, to-day, sitting up. The foot distorted and erysipelatous; applied tinct. iodine over surface. Prescribed tr. fer. mur. gtt. xx., quinia gr. j., morphiæ sulph. gr. ¼, *ter die.* Placed foot loosely in the fracture box and enjoined perfect rest.

*Sept. 1.* Patient was sitting up, contrary to directions, and, considering herself well, declined further treatment.

*Sept. 13.* Was sent for at noon, but being absent from home, did not see patient till 10 P.M. Found her in bed, suffering considerable pain; two openings in the skin corresponding to the piece of displaced bone; a free discharge of pus from each opening. She is suffering from an attack of asthma, to which she is subject. ℞. Morph. sulph. gr. ¼, chloroformyl. gtt. v., every hour till sleep is produced. A tablespoonful of port wine three or four times a-day. Bread and water poultice to foot.

*Oct. 3.* The bone being loose, and evidently causing much irritation, an incision was made on it and the bone removed. A copious discharge of pus took place at the same time.

*Oct. 17.* An abscess has formed on the dorsum of the foot, giving considerable pain. A surgeon, who had been called in during my absence, advised amputation. By pushing a small probe-pointed knife from the wound in the direction of the abscess, the pus was evacuated with immediate relief.

From this time, the case progressed favorably and without a bad symptom, and at the time of her confinement, in January of the present year, she could walk about her house with but little inconvenience, and entirely without pain.

